# Eco-Friendly Pavements Manufactured with Glass Waste: Physical and Mechanical Characterization and Its Applicability in Soil Stabilization

**DOI:** 10.3390/ma13173727

**Published:** 2020-08-24

**Authors:** M. Isabel Más-López, Eva M. García del Toro, Alfredo Luizaga Patiño, L. Jaime Marco García

**Affiliations:** Escuela Técnica Superior de Ingeniería Civil, Universidad Politécnica de Madrid, 28040 Madrid, Spain; mariaisabel.mas@upm.es (M.I.M.-L.); martin.luizaga@upm.es (A.L.P.); luisjaime.marco@upm.es (L.J.M.G.)

**Keywords:** sustainability, glass powder, binder, eco-friendly pavement

## Abstract

The use of glass waste, which by its nature cannot be recycled, might be a viable alternative in the manufacture of cements and concrete that is also economical and environmentally friendly. This alternative can reduce landfill areas with this inert residue but also limit the use of raw materials employed in the manufacture of cement and concrete and, consequently, contribute to minimize the environmental impact generated by this activity. In this research, the feasibility of using a limestone-type material treated with a binder manufactured with micronized glass powder and basic reagents, in the preparation of a gravel–cement- or soil–cement-type material, was analyzed. For this purpose, the strength, compactability, structural capacity, resistance to the action of water, stiffness and durability of the material obtained were characterized. From the tests that were carried out and the results obtained, it can be concluded that the use of glass powder, with a particle size of 16 μm, is ideal for the production of a gravel–cement- or soil–cement-type material. This material could be used as an environmentally-friendly pavement, especially suitable for peri-urban roads and park roads, where it can be used without coating, or as a base layer or sub-base for road surfaces, with little cracking due to shrinkage.

## 1. Introduction

The rapid population growth in the past few decades has significantly increased the type and quantity of both industrial and domestic waste. These wastes can be classified into two main groups: biodegradable waste and non-biodegradable waste [1]. Most waste materials that are not biodegradable are deposited in controlled landfills [1,2,3]. This waste will remain in the environment for decades, thus contributing to an important environmental problem: its elimination. However, the environmental impact that these wastes produce can be minimized, since many of them can be recycled or reused for certain applications, thus contributing to the circular economy [4]. Thus, integrated and optimized management of the waste is one of the pillars of the Sustainability Development Goals (SDG) [5], and it is absolutely necessary from the perspective of protecting the environment [6]. For this reason, it is essential to find applications that include more recycled material, thereby reducing the amount of waste requiring effective removal and preservation of natural resource [7].

Considering these economic and environmental concerns, it is worth highlighting glass waste among the various urban solid wastes. Due to its physicochemical characteristics, glass is very easy to recycle [8,9]. All types of glass waste that are obtained from the selective recovery of containers and packaging from the glass and ceramic industry are used in the recycling process [10,11]. 

At present, there are many studies that show the good qualities of glass waste to be reused as a substitute for certain materials in the manufacture of mortars and concretes. Glass waste was considered as one of the most suitable substitutes for sand and cement, due to its physical characteristics and chemical composition [10,12]. This reuse of waste material becomes a viable strategy to reduce the use of Portland cement and natural aggregates in the manufacture of mortars and concretes, thus reducing environmental impact, including a significant reduction of CO_2_ emissions, one of the gases responsible for the greenhouse effect [2,13], and the decrease of surface destined for controlled landfills [14,15]. Below are some of the properties presented by cements and concretes manufactured with glass dust residues:

(1) Improved workability and less water absorption as well as drying shrinkage and durability properties due to impermeability and the smooth surface that confers glass to mortars and concrete made from glass debris [16,17,18].
(2) Improved durability based on its resistance to abrasion and acid attack [19].
(3) Increased resistance to compression and elastic modulus after their exposure to high temperatures due to the fusion of the glass and filling of the cracks and pores [20].
(4) Reinforcement of photocatalytic activity due to its light transmitting properties [12].
(5) The leachates emitted when this type of cement material is used in the stabilization of soils are perfectly compatible with the environment [21].

All these properties, as well as the positive environmental impact generated by the use of glass waste in the manufacture of cement and concrete, have been widely studied [22]. In addition, several of these studies have shown that the decisive factor for avoiding alkali-silica reactions (ASR) is the granulometry of the glass waste used in the preparation of these products [23,24,25]. 

Idir et al. [25] specified that, with a granulometry of between 0.9 and 1 mm and with 20% substitution of cullet, the classic contractions due to the ASR do not occur. Corinaldesi et al. [24] went further and confirmed that a percentage of substitution of aggregates by glass powder greater than 70% can be reached, provided that the glass particles used have a size of between 36 and 50 μm. This shows that the decrease in the size of the glass particles used increases the pozzolanic properties of the manufactured binders [24,25].

One of the traditional uses of cements is the stabilization of soils that have unfavorable characteristics for use in civil engineering works. Soil stabilization is a method designed to improve the quality of the properties of the soil to be treated according to its characteristics and the objectives sought [26]. There are several methods to adjust, stabilize and improve the parameters of soil conditions, depending on the starting characteristics of the soil and the use of the soil [26].

One of the most common procedures is to stabilize the soil with cement. Once the cement is added to the ground and after setting, we obtain a material with a certain degree of cohesion and with a certain mechanical resistance. Two fundamental types of soil stabilized with cement are considered [27]: S-EST 3 (Stabilized Soil Type 3) with seven-day compressive strength of 1.5 MPa and soil cement with seven-day compressive strength of 2.5 MPa.

Dupas and Pecker [28] studied the static and mechanical properties of soil stabilized with cement. They observed that the addition of 5% Portland cement (PC) assumed an increase in the adherence conditions of sandy soils to 200–300 kPa. Later, Kukko [29] analyzed and compared the use of different cementitious materials, such as slag from blast furnaces and fly ash, to improve the resistance of several soils that presented different characteristics and concluded that the resistance of stabilized soils depended, above all, on the content of cement materials used.

Given the known capabilities of using glass powder as a binder, Wartman et al. [30] carried out laboratory studies to evaluate the feasibility of using glass dust to improve the characteristics of certain materials that are problematic for structures in civil engineering works, such as kaolin and quarry fines. They concluded that the friction force of fine grain soils increased considerably with the addition of glass dust and, thus, suggested that this material could be used to improve the properties of other materials considered marginal.

Grubb et al. [30,31] extended Wartman’s studies and concluded that the addition of glass dust in the stabilization of soils constituted by degraded materials greatly improved the properties of these soils: they reduced the water content and organic matter and decreased their plasticity index, besides being much more economic and environmentally friendly compared with the use of other methods such as Portland cement.

In previous studies carried out by García del Toro et al. [32], the good disposition of cementitious substances made with glass powder for soil stabilization was made evident. This study indicated that the gels formed by glass powder in the setting of the cementitious material had a special property of self-repair of soils stabilized with them.

In this work, we present the study of a limestone-based material treated with a binder composed of basic reagents and micronized glass powder to achieve a pavement that can be used in civil engineering works due to its physical and environmental characteristics and without the need to add a subsequent coating layer. 

## 2. Materials and Methods 

In this section, we describe the different materials that were used in the manufacture of the specimens to determine the characteristics of the pavement under study. 

### 2.1. Characterization of Materials

#### 2.1.1. Aggregate

A limestone aggregate was used. To establish its characterization, granulometry tests were carried out according to the UNE-EN 933-1 standard (a norm from the Spanish Association for Standardization or UNE, as per its Spanish abbreviation) [33]. The results are shown in Table 1.

#### 2.1.2. Cement

The cement used was a commercial Portland cement (CEM I 52.5 R), obtained from the Cementos Portland factory in Chinchón (Madrid). It had a volumetric mass of 3.12 g/cm^3^, a specific surface of 4440 cm^2^/g and a greenish-gray color. The proportion of particles with a diameter less than 8 μm was 41.5%; 99.7% of the particles had a diameter smaller than 96 μm. The chemical composition was as follows: CaO (65%), SiO_2_ (19%), Al_2_O_3_ (5.5%), Fe_2_O_3_ (2.65%), SO_3_ (2%), MgO (2%), Na_2_O (0.15%) and K_2_O (0.7%). Additional properties of the cement included loss due to fire (2%) and insoluble residue (1%).

#### 2.1.3. Glass Powder

The physical and mechanical characteristics of the glass waste used were obtained by different analytical techniques: laser granulometry (COULTER LS 100 Q, Oberkochen, Germany), X-ray diffraction observation (OLYMPUS, Hamburg, Germany) and scanning electron microscope (ZEISS EVO 10 SEM, Oberkochen, Germany). It should be noted that all the ground glass powder cited comes from the same batch of waste. 

The glass waste was turned into ground glass powder with different granulometries in order to compare the effect of size on the physical and mechanical properties of the cementitious material. The classification of the glass was carried out in different phases.

##### Preliminary Sampling

**Figure 1:**
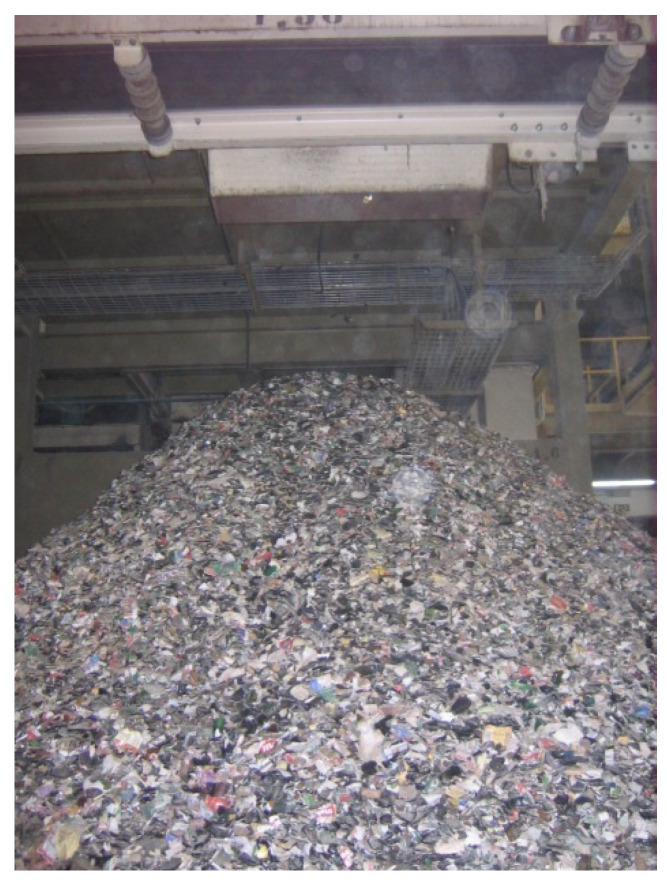
Residues of optical classification.

All tests were carried out with the same batch of waste selected by optical classification which, in addition to the presence of refractory elements, was characterized by including a high proportion of paper, plastics and non-ferrous metals (Figure 1). The selected batch of waste could contain particles of glass of any color, i.e., white, green, brown and blue (glass by different color was not separated).

Although the largest size of the glass waste particle was 40 mm, the most important feature to define the means for grinding is its thickness (1–3 mm), since the pieces of glass are usually long and narrow.

An analysis of the content of glass and other wastes was carried out on three dry residue samples of 20 ± 0.01 kg, resulting in the following proportions by weight of the sample: glass, 90.7%; paper, 4.6%; cork, metal and plastic plugs, 1.3%; and ceramic, 3.4%.

It was observed that the glass represented most of the residue analyzed. However, although paper only represented 4.6% of the total mass of the sample, its volume is important. To remove all the previously mentioned impurities, the waste glass was rinsed with water and after dried at 135 °C to reduce the moisture content.

##### Composition

The main chemical compounds found in the glass waste used in the manufacture of cementitious material were: 71% SiO_2_, 11.80% Na_2_O, 0.60% K_2_O, 11.28% CaO, 1.40% MgO, 2.20% Al_2_O_3_, 11.60% Fe_2_O_3_, 0.07% TiO_2_ and 0.05% P_2_O_5_.

##### Grinding

**Figure 2:**
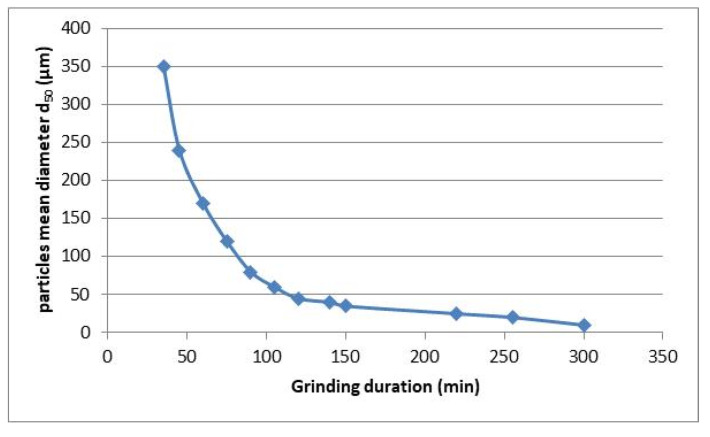
Evolution of the size of the glass particles as a function of the grinding duration of 20 kg of glass waste inside the bar mill.

The residues from the optical classification were ground using a bar mill equipped with 15 bars of three different diameters. During grinding, in different time intervals, samples were taken to test the kinetics of the process (Figure 2). The quantities extracted were small enough so that they did not affect the behavior of the mill but large enough so the results obtained in the laser granulometry tests were representative of the kinetics. Then, the same weight of waste (20 ± 0.01 kg) was introduced in each milling test to reproduce the same working conditions, and the granulometry of the glass powder obtained was checked and represented as a function of the operating time of the mill.

The reduction of the size of the particles is increasingly difficult to achieve as grinding progresses. This translates into an increase in energy required to obtain powder with a very small granulometry and, consequently, a high economic cost when this process is carried out during at industrial scale. Therefore, it is necessary to find the balance between the cost and the efficiency of grinding.

##### Granulometric Characterization of Glass Powder

Three lots of milled waste were made, varying for each the duration of the milling process. In this way, three samples of glass powder with different granulometries were obtained.

The characterization of the glass powders is given by three characteristic dimensions, namely d_10_, d_50_ and d_90_, which represent a diameter measurement for which 10%, 50% and 90% of the particles, respectively, have a diameter below the measurement used as a reference. For the study, the d_50_ value was used for the characterization of the different batches of ground glass.

The samples were analyzed in the COULTER LS 100 Q laser granulometer (Oberkochen, Germany) with a cell obscuration coefficient between 8% and 12%. Table 2 shows the granulometric characteristics, (referred to the average of 10 tests), of the three samples used with different grinding times.

**Figure 3:**
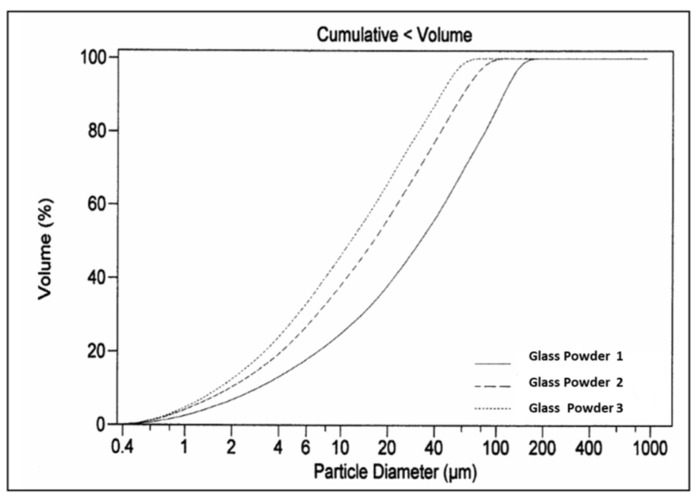
Distribution of the accumulated granulometry of the three batches of glass powder.

**Figure 4:**
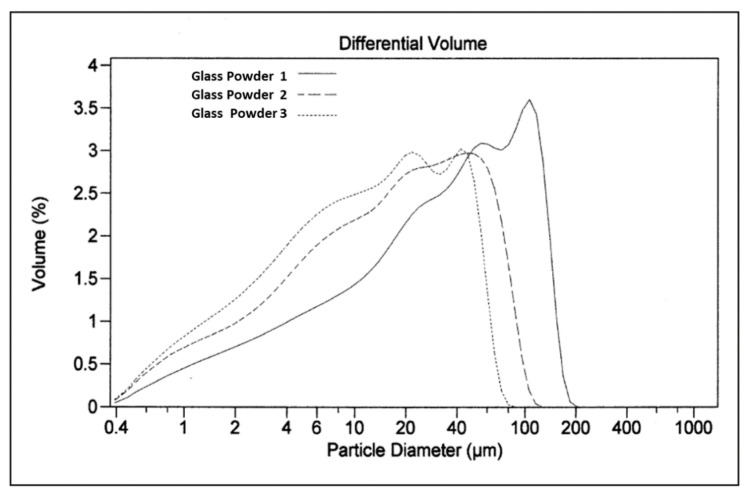
Differential particle size distribution of the three batches of glass powder.

The cumulative granulometric curves of the three samples (Figure 3 and Figure 4) reveal considerable differences in the distribution of particle size.

In the graph of the differential granulometric distribution, it can be observed that prolonged milling reduces the maximum granulometry limiting the production of fines. On the other hand, a continuous granulometric distribution is observed, obtaining a single group of particles (Figure 4).

##### X-ray Diffraction

**Figure 5:**
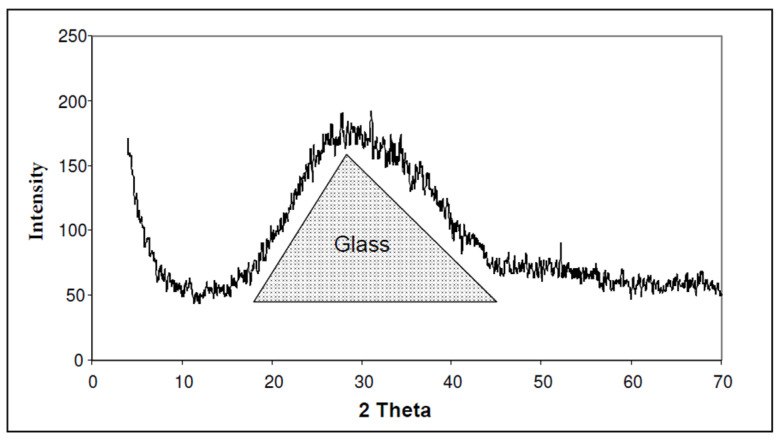
X-ray diffractogram of the glass powder of d_50_ = 33 μm. The dotted line shows the bulge of the baseline indicating the presence of amorphous phases.

Glass subjected to X-ray diffraction tests (Figure 5) results in a wide diffraction band between 15 and 45 degrees, which corresponds to its amorphous and disordered structure.

##### Characterization in the Scanning Electron Microscope

**Figure 6:**
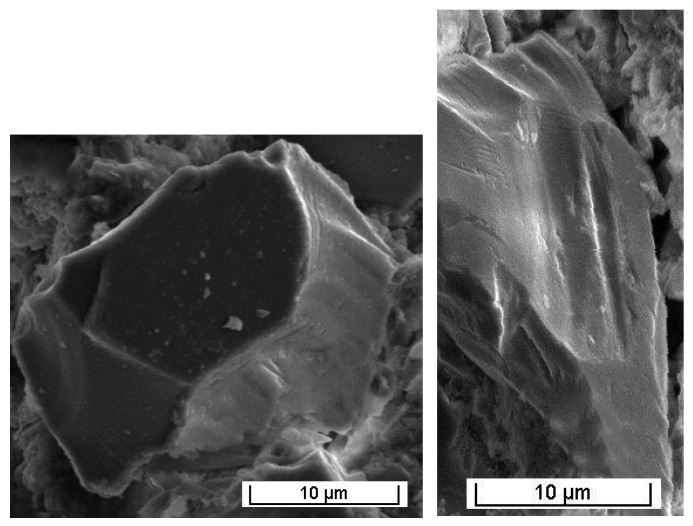
Views of a glass particle (MEB in secondary electrons).

From observations done through a Scanning Electron Microscope (SEM) (Figure 6), glass grains between 1 and 10 μm in size can be seen. These grains show acicular shape and conchoidal fractures. It should be noted the absence of fine elements glued to these glass particles and their low porosity.

##### Volumetric Mass of Glass Powders

The absolute volumetric mass for each of the batches of glass powder was measured with the following results: T_1_d_50_ = 2.53 ± 0.01 g/cm^3^, T_2_d_50_ = 2.54 ± 0.01 g/cm^3^ and T_3_d_50_ = 2.55 ± 0.01 g/cm^3^. 

### 2.2. Tests and Working Plan

The first step of the tests was to obtain the ideal mixture based on pozzolanic binder material mainly prepared with glass powder and Portland cement. In the preparation of the pozzolanic binder, two fundamental issues had to be taken into account: the granulometry and the percentage of glass powder used.

In this investigation, glass powder of d_50_ equal to 16 μm was used, which offers interesting mechanical results with an energy cost clearly lower than that necessary to obtain smaller granulometries of the glass powder. This material with these specifications is more useful in the field of Civil Engineering [13]. According to Corinaldesi et al., the percentage of suitable glass powder, to avoid ASK reactions, should be 70–90%. [24]

Once the pozzolanic binder was obtained, mixtures with different proportions were tested. It was observed that the optimal results were obtained for the proportions of 84.68% limestone aggregate, 8% pozzolanic binder and 7.32% water.

To carry out the tests, cylindrical specimens of 15.24 cm in diameter and 17.80 cm in height were prepared. The preservation and curing of the specimens were carried out following standard Norms of the Spanish Laboratory of Transport (NLT, as per its Spanish abbreviations)-310/90 [34] in a standard chamber at 20 ± 2 °C.

The following laboratory tests were then performed on the mixtures described above:

(1) Granulometry of the aggregate used according to UNE-EN-933-1: 2012 Second bullet [33].
(2) Atterberg limits according to NLT 105-98 [35], UNE 103-103-94 [36].
(3) Compactability through the modified Proctor compaction test, according to UNE103501 standard [37].
(4) The support capacity, through the California Bearing Ratio (CBR) following UNE 103502 [38].
(5) The structural capacity, through the compression resistance test (at different ages of the specimens), according to the standards NLT-304 [39], NLT-305 [40] and NLT-310 [34].
(6) The resistance to the action of water, through the resistance test by immersion in water (immersion–compression), according to NLT-162 [41].
(7) The stiffness test by testing Dynamic Modules (at different ages of the specimens), according to the NLT-349 standard [42].
(8) The durability of the pavement laid with the treated material, regarding the action of the weather agents, was studied by tests of loss of mass of test pieces subjected to cycles of humidity-dryness and freeze-thaw cycles according to the standards NLT-302 [43] and NLT-303 [44], respectively.
(9) The durability of the pavement laid with the treated material, regarding resistance to traffic loads, was estimated by means of an analytical calculation made from the mechanical properties measured in the tests mentioned above.

## 3. Results

### 3.1. Limits of Atterberg

The Atterberg limit test was conducted according to NLT 105-98 [35] and UNE 103-103-94 [36] standards. The results obtained are shown in Table 3:

The test indicates that the plasticity index corresponds to a non-plastic material, since it complies with the specifications collected by the NLT 105-98 [35] and UNE 103-103-94 [36] standards in this regard.

### 3.2. CBR Test

The CBR Test was carried out following the guidelines indicated by the standard UNE 103502 [42] and the results obtained are reflected in Table 4:

The medium CBR support capacity at seven days was 266, which is higher than the minimum required following the standard used to carry out the test for a stabilized soil type S-EST 2, established at 12 as a minimum, and of the same order as the values usually presented in artificial gravel soils, i.e., 100–300 as a minimum [45]. The swelling reflected in the tests is very small, thus swelling in the tested pavement is not expected. 

### 3.3. Modified Proctor

The modified proctor test was carried out according to UNE 103-501-194 [37] with the characteristics that are reflected in Table 5 by means of automatic compactor “Compactester”.

**Figure 7:**
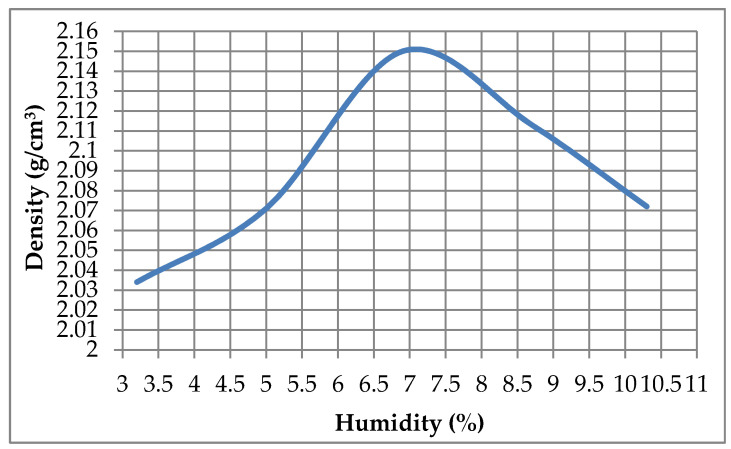
Modified proctor.

The results of the test are represented in Figure 7.

In the results obtained for the modified proctor, it can be seen that the maximum density of compaction was 2.15 g/cm^3^ with an optimum humidity of 7%. These results show a notable improvement in terms of density compared to soils treated with conventional cements [27].

### 3.4. Compression Resistance

**Figure 8:**
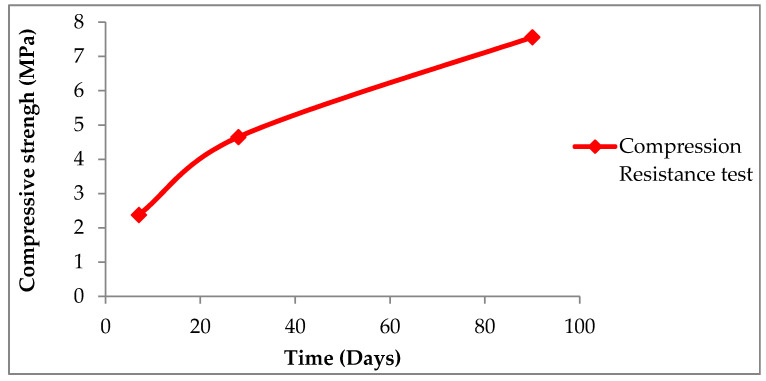
Compressive strength.

The standard used for the compressive strength test was NLT-304 [43]. The results obtained are represented in Figure 8.

At the age of seven days, a compressive strength of 2.38 MPa was obtained. The resistance to compression at the age of 28 days is almost double than that obtained at seven days (an increase of 94%), and that obtained at 90 days is more than three times that corresponding to seven days. This value meets the specifications established for a stabilized soil S-EST 3 (minimum of 1.5 MPa) and for a cement floor, considering the pozzolanic binder manufactured with powder micronized glass, as a special cement (values included in the interval 2.13–3.83 MPa). Resistances obtained at 90 days were of the same order of magnitude as those that generally have a soil–cement built with soils of adequate quality [45]. The results obtained in the compressive strength test indicate that this material is suitable for the purpose studied.

### 3.5. Immersion–Compression Resistance Test

The resistance test to the action of water at the age of 90 days was carried out using the NLT-162 standard. 

**Figure 9:**
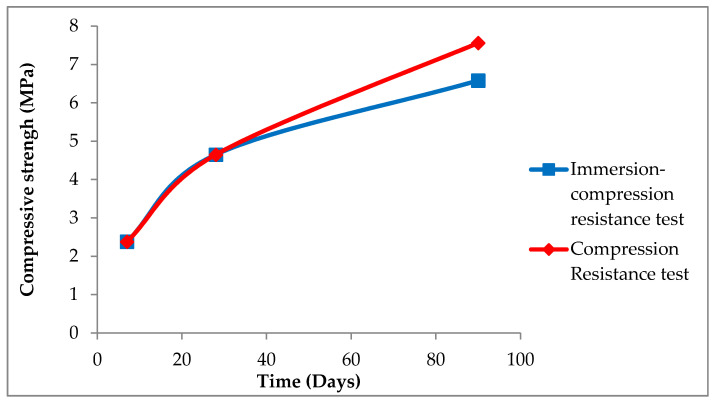
Comparison of compressive strength and compression-immersion graph results.

As can be seen in Figure 9, after the immersion test, only a 23% loss of resistance was observed with respect to that seen in the same period of time (90 days) for specimens that were not subjected to the immersion test. That is, the resistance was 87% of the initial resistance, which can be considered high [45].

### 3.6. Stiffness Test

The rigidity test was carried out by means of the dynamic modules test (at different ages of the test pieces), according to the NLT-349 standard. A dynamic module for the age of 90 days of 19,000 MPa was obtained, with a value higher than that usually obtained in a cement floor (8000–10,000 MPa) and very close to that of a gravel–cement (20,000–25,000 MPa) [45]. This indicates that, taking into account the technical prescriptions required in the standard used to carry out the test, the material studied is suitable for the use proposed in this study.

### 3.7. Durability Test—Action of the Weather

Tests of mass loss in research samples subjected to humidity–dry cycles and freeze–thaw cycles were carried out according to standards NLT-302 and NLT-303, respectively.

The results of the test are as follow: the loss of mass in the tests of humidity–dryness and freeze–thaw cycles for specimens cured for seven days was 2.6% and 3.7%, respectively. In similar materials composed by sand and 5–6% cement, in specimens cured for the same period of time, losses were obtained in the moisture–dryness test of 1–5% and in the freeze–thaw test of 4–7%, showing that the durability against the action of the weather agents was similar to those obtained for materials with similar granulometry and manufactured with cement. For this reason, regarding this test, the material under study would also meet the specifications required by the standard used in the durability test.

### 3.8. Durability Test—Resistance to Traffic Loads

After conducting the laboratory tests, an analysis of resistance to road traffic was carried out. This analysis was done using an analytical model. From the analytical calculations of soil made using this material and with a gravel base of more than 20 cm thickness, resting directly on an esplanade of CBR 5, the results indicate 10 cm are suitable for light traffic of cars and of 15 cm for traffic of very light commercial vehicles (2 t/axle). With irregular heavy traffic, this paving material seems more suitable as a base layer, in thicknesses greater than those indicated in the previous paragraph, and with a bituminous tread layer.

In view of the results obtained, and taking into account that all of them comply with the specifications required by the standards used in conducting the tests, it can be stated that the material subject of study, a cementitious material manufactured with glass waste, could be used as an eco-friendly alternative in soil stabilization. Because of the physical and mechanical characteristics of the research material, it could be seen as an optimal product for the construction of peri-urban roads and park roads, when an uncoated environmental purpose is required. By not being used on the upper surface, it can release a thin layer of aggregate that provides a feeling of flexibility for users, ideal for sport applications where the impact on the ground plays an important role.

The environmentally-friendly properties of the use of glass in this paving material must also be emphasized, even from an esthetical point of view, since it minimizes the landscaping impact that traditional conglomerates would cause and also makes the color of the roads stable and perfectly integrated into the landscape, since the soil will acquire the color of the aggregate used.

## 4. Conclusions

The use of glass waste in the preparation of cements and concretes has been extensively studied. The aim of this research was to evaluate the physical and mechanical characteristics of a cement prepared with a pozzolanic binder made of glass waste, in order to confirm its suitability as a paving material. The following proportions were found optimal when preparing the material under study: 84.68% limestone aggregate, 8% pozzolanic binder made of glass waste, with a particle size of 16 µm, and 7.32% water. To characterize the resulting material, the following tests were performed: granulometry of the aggregate, plasticity index, compactability, support capacity, resistance to the action of water, structural capacity, stiffness and durability tests. The tests were conducted according to Spanish regulated standards. Based on the results obtained, it can be concluded that the material obtained with the use of glass waste as a pozzolanic binder met or exceeded the specifications required for a traditional cement employed as a pavement material and, in addition to that, the use of glass powder as a starting material reduces the amount of waste, hence contributing to environmental conservation.

## Figures and Tables

**Table 1:** Aggregate granulometric analysis.

Aperture	Retained among Tamices			
UNE (mm)	Weight (g)	% Partial	% Accumulated	%Pass into Total Sample
100	-	-	-	-
80	-	-	-	-
63	-	-	-	-
50	-	-	-	-
40	-	-	-	-
31.5	-	-	-	-
25	-	-	-	-
20	-	-	-	-
16	-	-	-	-
8	-	-	-	100.0
4.0	282.0	9.9	9.9	90.1
2.0	789.0	27.8	37.8	62.2
1.0	503.0	17.7	55.5	44.5
0.500	406.0	14.3	69.8	30.2
0.250	261.0	9.2	79.0	21.0
0.125	148.0	5.2	84.2	15.8
0.063	62.0	2.2	86.4	13.6

Particle Size Distribution.; sample: fine Greek arid; test method: washing and sieving.

**Table 2:** Granulometric characteristics of glass powders produced as a function of grinding time.

Glass Powder Used	Grinding Duration	d_10_	d_50_	d_90_
T1	2 h 30 min	2.92 ± 0.01 µm	33 ± 1 µm	110 ± 3 µm
T2	4 h 15 min	1.96 ± 0.01 µm	16 ± 1 µm	59 ± 2 µm
T3	5 h	1.65 ± 0.01 µm	11 ± 1 µm	43 ± 2 µm

**Table 3:** Limits of Atterberg.

Liquid Limit	Plastic Limit	Plasticity Index
34.5	19.4	15.1

**Table 4:** Summary CBR test results.

Specimen	CBR Index	Swelling (%)	Density (g/cm^3^)
1	163	0.09	1.975
2	284	0.09	2.101
3	350	0.09	2.163

**Table 5:** Modified Proctor test characteristics.

Characteristics	
Mold volume (cm^3^)	2320
Compaction hammer (kg)	4.535
Fall height (cm)	45.7
Weight of material used (kg)	24
Number of layers	5
Number of hits per layer	60
